# Family resilience: perception of family members of psychoactive substance dependents*

**DOI:** 10.1590/1518-8345.3816.3449

**Published:** 2021-06-28

**Authors:** Bianca Oliveira Ruiz, Sonia Regina Zerbetto, Sueli Aparecida Frari Galera, Bruno José Barcellos Fontanella, Angélica Martins De Souza Gonçalves, Simone Teresinha Protti–Zanatta

**Affiliations:** 1 Universidade Federal de São Carlos, São Carlos, SP, Brazil.; 3 Universidade de São Paulo, Escola de Enfermagem de Ribeirão Preto, PAHO/WHO Collaborating Centre for Nursing Research Development, Ribeirão Preto, SP, Brazil.; 4 Universidade Federal de São Carlos, Departamento de Medicina, São Carlos, SP, Brazil.

**Keywords:** Psychological Resilience, Family, Family Relations, Substance-Related Disorders, Psychological Adaptation, Psychiatric Nursing, Resiliência Psicológica, Família, Relações Familiares, Transtornos Relacionados ao Uso de Substâncias, Adaptação Psicológica, Enfermagem Psiquiátrica, Resiliencia Psicológica, Familia, Relaciones Familiares, Trastornos Relacionados con Sustancias, Adaptación Psicológica, Enfermería Psiquiátrica

## Abstract

**Objective:**

to understand the perception of family members of psychoactive substance dependents on the elements of the functioning of their family in family resilience.

**Method:**

a qualitative approach study, based on the theoretical interpretive framework of family resilience from a systemic perspective. The participants were eleven family members of psychoactive substance dependents from a Psychosocial Care Center - Alcohol and Drugs, from a city in the state of São Paulo. For data collection, semi-structured interview, genogram and ecomap were used. Data analysis was based on the Content Analysis technique, thematic category.

**Results:**

from the interviews, three thematic categories were formulated: mobilization in search of support and social support; positive perspectives that would strengthen the family, and assertive communication. These categories point to references to the mobilization and unity of the family in search of social support in the intra-family, extended family and extra-family contexts and positive perspectives, such as persistence, perseverance, hope, faith and religiousness.

**Conclusion:**

the situation of having a psychoactive substance dependent in the family seemed to mobilize coping devices and attempts to overcome them through the resilience forces. The results may favor the daily clinical reasoning of the health professionals, helping them to recognize and value the identified resilience attributes.

## Abstract (pt)

**Objetivo:**

compreender a percepção dos familiares de dependentes de substâncias psicoativas sobre os elementos do funcionamento de sua família na resiliência familiar.

**Método:**

estudo de abordagem qualitativa, fundamentado no referencial teórico interpretativo de resiliência familiar na perspectiva sistêmica. Participaram onze familiares de dependentes de substâncias psicoativas de um Centro de Atenção Psicossocial - Álcool e Drogas, de uma cidade do interior paulista. Para a coleta de dados, utilizou-se entrevista semiestruturada, genograma e ecomapa. A análise dos dados baseou-se na técnica de Análise de Conteúdo, categoria temática.

**Resultados:**

a partir das entrevistas, foram formuladas três categorias temáticas: mobilização em busca de apoio e suporte social; perspectivas positivas que fortaleceriam a família, e comunicação assertiva. Essas categorias apontam referências à mobilização e união da família em busca de suporte social nos contextos intrafamiliar, de família ampliada e extrafamiliar e perspectivas positivas, como persistência, perseverança, esperança, fé e religiosidade.

**Conclusão:**

a situação de ter um dependente de substâncias psicoativas na família pareceu mobilizar dispositivos de enfrentamento e tentativa de superação por intermédio das forças de resiliência. Os resultados podem favorecer o raciocínio clínico cotidiano dos profissionais de saúde, ajudando-os no reconhecimento e na valorização dos atributos de resiliência identificados.

## Abstract (es)

**Objetivo:**

comprender la percepción de los familiares de dependientes de sustancias psicoactivas sobre los elementos del funcionamiento de su familia en la resiliencia familiar.

**Método:**

estudio cualitativo, basado en el marco teórico de la resiliencia familiar en la perspectiva sistémica. Participaron once familiares de dependientes de sustancias psicoactivas de un Centro de Atención Psicosocial - Alcohol y Drogas, de una ciudad del interior de San Pablo. Para la recolección de datos se utilizaron entrevista semiestructurada, genograma y ecomapa. El análisis de datos se basó en la técnica de Análisis de Contenido, categoría temática.

**Resultados:**

a partir de las entrevistas, se formularon tres categorías temáticas: movilización en busca de apoyo y soporte social; perspectivas positivas que fortalezcan a la familia y comunicación asertiva. Estas categorías hacen referencia a la movilización y unidad de la familia en busca de apoyo social en los contextos intrafamiliares, la familia extendida y extrafamiliar y perspectivas positivas, como persistencia, perseverancia, esperanza, fe y religiosidad.

**Conclusión:**

tener un dependiente de sustancias psicoactivas en la familia pareció movilizar dispositivos para afrontar e intentar de superar la situación a través de la fuerza de la resiliencia. Los resultados pueden favorecer el razonamiento clínico diario de los profesionales de la salud, ayudándoles a reconocer y valorar los atributos de resiliencia identificados.

## Introduction

The use of psychoactive substances (PAS) tends to have a negative impact on various aspects of the life of the dependent person and their family – family conflicts, domestic violence and changes in interpersonal relationships are common, for example^(^ 1 ^-^ 2 ^)^.

National and international studies on family and PAS dependence usually approach the family as a risk factor or setting; therefore, a negative view of the dysfunctionalities of family relationships predominates^(^ 2 ^-^ 3 ^)^.

However, functional aspects of family dynamics have been evidenced in the context of dependence on PAS^(^ 4 ^-^ 5 ^)^. In this view, it is constructed, developed and lived in a relational context and experience throughout life. Thus, family resilience, perceived in the family unit and its functionality, considers the way in which this group faces and deals with adverse situations, to reorganize itself effectively and to overcome such difficulties^(^ 6 ^)^.

International studies on family resilience point out that the families that experience situations of psychological distress reorganize their functional patterns by establishing daily routines, promoting regular events that minimize or alleviate stressful situations^(^ 6 ^)^, by providing intra- and extra-family connections^(^ 6 ^)^ and by seeking support resources and intra- and extra-family social support^(^ 6 ^-^ 7 ^)^. In Brazil, there are few studies on the theme of family resilience^(^ 8 ^)^ and, in the context of mental health, the focus involves characteristics and individual resilience factors of the family members, which can influence another family member^(^ 8 ^)^.

National studies on resilience and consumption of PAS involve quantitative methods, with the application of scales with dimensions of family functioning to investigate the relationship between family resilience and problems arising from families that consume alcohol and other substances^(^ 9 ^)^, as well as validating a scale that involves dimensions of family functioning and resilience^(^ 10 ^)^. Both studies point to the recognition of resilience as a protective factor for the family members who experience problems with PAS^(^ 9 ^-^ 10 ^)^. Such studies emphasize that experiencing situations of high vulnerability can awaken individual positive aspects that help the person to face and overcome adversities^(^ 9 ^-^ 10 ^)^.

Thus, in 2013, the World Health Organization developed the Global Mental Health Plan - 2013-2020, stressing that the mental health of a population requires people to recognize their potential to deal with life’s conflicts and tensions. Therefore, investing in studies that understand the adverse situations experienced by the family members of the PAS dependent can be useful to implement strategies to promote mental health that include the family.

Understanding the family’s resilience process, from the perspective of the family member, will contribute to deepen the knowledge about the concept of resilience and its applicability to the Nursing practice. It is understood that such a practice consists of planning strategic Nursing care actions with the families, providing them with the tools to develop and strengthen their resilience, enabling them to respond as a functional unit.

Given the above, the questions that guided this research were: How have the family members of PAS dependents faced and overcome this problem? What coping strategies are used by these individuals and recognized by them as keeping them in resilience? What elements of family functioning contribute to the family resilience process, from the perspective of the family members? What are the forces recognized by these members that cause positive changes in the functioning of their family?

The objective was to understand the perception of the family members of psychoactive substance dependents on the elements of the functioning of their family in family resilience.

## Method

A qualitative approach study carried out with family members of PAS dependents treated at a Psychosocial Care Center - Alcohol and Drugs (Centro de Atenção Psicossocial - Álcool e Drogas, CAPS-AD) in a city in the inland of São Paulo, Brazil. In this study, Walsh’s concept of family resilience^(^ 6 ^)^ was adopted, which values the way in which the family system faces adverse and challenging experiences, reorganizes itself efficiently and overcomes them, resonating such impacts in the entire family and in their relationships^(^ 6 ^)^.

The constituent elements of the concept of family resilience involve the belief system, organizational patterns and communication processes. Beliefs are related to the family’s perceptions and understanding of the world. The family unit can extract positive meanings from an adverse situation to face and overcome it, through attitudes of perseverance and hope, in order to identify its strengths and its potential to solve problems^(^ 6 ^)^. The family can also use its religious and spiritual resources, such as faith, meditation and religious practices, as well as learning from adversity, which are important sources of resilience^(^ 6 ^)^.

Family organization is understood by the organizational pattern that promotes an effective relationship. The strengthening of resilience results from the flexible functional structure of the family, in which connection, cooperation and support occur between family members and the discovery and mobilization of their social, economic and labor resources^(^ 6 ^)^.

The communicative process consists of ways of communication that facilitate the functioning of the family^(^ 6 ^)^, which must be clear, enlightening, and enablers of the open expression of emotions and sharing of feelings, in order to decide and solve conflicts collectively^(^ 6 ^)^.

Eleven family members of PAS dependents indicated by the CAPS-AD health team participated in the study: a sister, a brother-in-law, a grandmother, four mothers and four fathers.

The inclusion criteria were the following: being a family member of a PAS dependent over 18 years of age; participating in family or individual care groups with health service professionals, considering these indicative of resilience, and living with the user at least twice a week. The exclusion criterion was being a family member with signs of PAS poisoning on the day of the interview.

Sampling was intentional and it was closed due to exhaustion, that is, when all the available and eligible families had been interviewed, according to the inclusion and exclusion criteria^(^ 11 ^)^.

The meeting was scheduled by telephone, constituting a meeting with each family. The interviews were conducted at the CAPS-AD or at the family’s home, from June to November 2016, with a mean duration of 1 hour and 30 minutes, conducted by two researchers (the first author, MS student, and the second author, PhD and university professor).

A semi-structured interview was conducted, using the genogram and ecomap^(^ 12 ^)^ for the initial approach of the interviewees, with the aim of facilitating interaction and welcoming the family. Such instruments were built manually during the interview and validated with the families, making it possible to understand the family configuration, the organizational and relational patterns in the family functioning considered positive and flexible, highlighting the fundamental processes for relational resilience, as well as the of social support network. Next, it was sought to carry out the sociodemographic characterization of the participants and to introduce the guiding question: "Tell me how your family has lived, faced and overcome the drug problem", from which the expression of the participant’s thoughts was stimulated.

The interviews were recorded, transcribed and analyzed according to the content analysis technique, thematic category^(^ 13 ^)^. The analysis process was carried out independently by two researchers, who met later to review, decide, validate and reach consensus on the reports with the meanings of the themes and categories. The process consisted of: reading attentive to the details of each interview, identifying and coding relevant phrases/paragraphs that pointed out the family’s perceptions of the situation experienced, the coping and overcoming strategies for the adversities, and the positive forces used in the organization and functioning of the family. Spreadsheets for each family were prepared in a Microsoft Word file, containing notes that indicated emerging themes and their respective illustrative reports. Afterwards, the researchers read all the worksheets and sought to group and regroup the themes and reports of all the interviews, which were constituted in nuclei of meaning (considering both the frequency of occurrences and the inference of their importance for the interviewees). Subsequently, the classification of themes made it possible to formulate thematic categories^(^ 13 ^)^. From this process, three thematic categories were constituted, commented on in the next section of this article, which were interpreted through the elements present in the functioning of families, which help in family resilience, according to the Walsh model^(^ 6 ^)^, organizational patterns, belief systems and communication patterns.

The excerpts that illustrate the participants’ reports were identified according to the following convention: F (family number) and an additional letter identifying the relationship [where M = Mother, P = Father ("*Pai*" in Portuguese), I = Sister or Brother ("*Irmã*" or "*Irmão*" in Portuguese), and C = Brother-in-law '("*Cunhado*" in Portuguese)]. Thus, the code F5-M means the interview with family 5, having interviewed the mother of the user with PAS dependence.

The study was approved on 10/29/2015 by the Committee of Ethics in Research with Human Beings, under opinion number 1,302,491. All the participants signed the Free and Informed Consent Form after clarifying the objectives and data collection technique.

## Results

Regarding the characterization of the family members, the degrees of kinship with the PAS dependent consisted of a sister, a brother-in-law, a grandmother, four mothers and four fathers, aged between 45 and 97 years old. Among the families, four are of a nuclear configuration. As for religion, there was unanimity of Christians, being a practicing evangelical family, while the others are also practicing Catholics.

Three thematic categories were formulated that describe aspects of the perception of the family members of PAS dependents about what they consider to be or happen in the functioning of their family and that assist them in what the theory calls family resilience: a) mobilization in search of support and social support; b) positive perspectives that would strengthen the family; and c) assertive communication.

In the first category, *mobilizing the family in search of support and social support*, family resilience consisted of the mobilization of emotional, affective, informational, institutional, economic and social resources by the families, in order to face and overcome the challenges experienced, both in the intra-family and extended family environment, and in the extra-family environment. Regarding the intra-family and extended family environment, there was a change in routines, with participation in mutual help groups and family groups in the CAPS-AD, to assist in the treatment of their relative and to help them live with them.

*I went yesterday, in "Amor Exigente", right? I went with his [dependent] wife because, as she’s working, she’ll need to attend meetings at night. So we went there [mutual aid group], but normally we’ve been coming to the CAPS since he was admitted.* (E1F1-I)

As a demonstration of concern and care, some family members gave up a job to accompany the dependent relative in the treatment.

*So we [couple] gave up everything [...]. That I gave up another salary, another job that I had, so that I could dedicate myself exclusively to her treatment [dependent].* (E1F2-P)

The union and the sense of family connection were also discussed. Strengthening affective bonds, establishing positive alliances and valuing feelings of love among the family members gave strength to the family unit, apparently helping in the process of overcoming the crisis.

*We [husband and wife] are very close. [...] We’re still together today (laughs). We support each other a lot. [...] In times of difficulty, then we became much more united than we already were.* (E1F3-M)

The challenging situations experienced by the family members allowed them to reflect on their relationships, changing their behavior and strengthening the couple’s bond to face the crisis situation.

*We [couple] hardly talked, be present, now we’re more present, more together [...]. We realized that we needed more unity to overcome this, and to believe in the strength to overcome and to move forward and strong. And we looked back and saw that we were quite apart, far from each other, there was little dialog. [...] We’ve been feeling much better, because unity, as the saying goes, "unity is strength"; so people coming together feel stronger to face things.* (E1F5-P)

A loving feeling, expressed and shared among the family members, was alleged as a reason for not giving up in the face of adversity.

*And love, right? That we have with the family, for the son, the love that we have with each other [...]. We need to have love too, because otherwise, we can’t.* (E1F5-M)

The importance of investing time in entertainment and leisure activities together was recognized to strengthen bonds and affective ties between family members, promoting moments of humor, distraction, relief from tension, relaxation and outbursts, as well as remembering significant moments in life.

*Sometimes we say [couple]: "Let’s have an afternoon coffee together". And he [interviewee’s brother] also comes, he passes by. So, between brothers, we’re very close. This afternoon coffee moment, we forget everything, right? We start laughing, telling stories, we relax. [...] We talk, laugh, tell a joke, right? [...] Both my family and his family are the same. [...] Make afternoon coffee. We have the same customs.* (E1F3-M)

As for the economic difficulties, financial support was sought from family members or they resorted to another job, reorganizing themselves in their schedules.

*Yes, difficulty [...]. It’s not cheap for you to keep someone in the clinic. The wife [of the dependent] was kind of in the hand. She has, she has a job, but she’s alone, so she had to find another job to add to this, reconcile, put her daughter in school all day so that she could have part-time work and reconcile with the bath and grooming, and we provide support, both as far as possible, psychological and financial. Now it’s normalizing.* (E1F1-I)

Finally, emotional support from other family members was resorted to, through listening and advice.

*Emotional. [...] we are family, we feel more comfortable talking, right? You feel safe talking to a brother or someone close to us, right? It’s hard to open up to an outsider, you don’t talk, do you? We talk, they advise, right? It gives that support to people, and everything will be fine. We’re more relieved, right?* (E1F3-M)

As for extra-family support, there was a search for community resources (recovery clinics, private hospitalizations or mediated by the CAPS-AD, public advocacy and mutual help groups: Amor Exigente and Narcotics Anonymous), health professionals and relatives or friends of the family. This seems to have helped to understand the situation, bringing new meanings.

*So, we looked for a rehabilitation clinic and he was admitted.* (E1F1-I)

*We [couple] are going to start in "Amor Exigente" too, me and her, and she [dependent] is dying to go to NA [Narcotics Anonymous].* (E1F2-P)

Through its professionals and family group, the CAPS-AD was recognized as institutional, informational and emotional support for the management of crisis situations, and this environment is a space referred to as learning and welcoming.

*So, first the CAPS-AD here, right? That they [professionals] help me a lot, the family group has helped me a lot.. [...] I like to participate. [...] We learned, we learn a lot, how to deal with him [dependent]. [...] when we come to the family group, we leave here renewed, we feel much better, you know, we feel the strength to continue facing, the person, the patient [...] so I feel so strong, strengthened to be able to face him at home [...] I leave here calmer, calmer.* (E1F4-M)

Another source of emotional support referred to the friends, whether in attention, spiritual care, words of comfort and encouragement.

*We [couple] seek strength [...] in the people who are there with us always giving support, there is always a word that a person says, already put you up there, you know, help, because if you take it alone, you fall down there. [...] word of comfort, is always there asking you how it is. So when the person gives you some strength, gives you a hug, gives you a friendly word, comforts you a little, comforts you.* (E1F5-M)

In the second category, that is, family resilience involving positive perspectives that strengthen the family, beliefs were reported that would have helped in resilience, such as the importance of maintaining persistence and perseverance.

*That we [couple] have to fight, you can’t give up, right? We have to go to the end, as they [professionals] say [...]. So we have that support, feel that strength to fight, don’t give up, right?* (E1F3-M)

Although negative feelings and emotions were exposed, faith and hope were reported that the dependent family member would recover, not returning to substance use and resuming old healthy habits and behaviors.

*You need to have faith, right, because if not, we can’t take it. It seems that we are creating strength in life more and more, and we are taking it. We can’t lose faith, we can’t lose hope, we can’t lose love, we can’t lose patience, we must always be there.* (E1F5-M)

*So, everything will be fine, that we think that when he leaves there [clinic], everything will be different. [...] We have this hope, right? [...] Hope that he won’t be using drugs anymore. That he’ll go back to work, study, right?* (E1F3-M)

For some, the attachment to religiousness and religious practices seemed important to acquire strength and courage in the management of the adverse and challenging situation of PAS, reported as one of the strategies of spiritual and emotional support, recognizing it as a motivating source to live and continue in treatment.

*I think that if it weren’t for him, we wouldn’t have the strength to be here today, right? It was very difficult for us. And we put the knee on the floor and pray, and ask and pray and do, right? For God to help, for God to lay hands, let everything change, right? Change his mind, right?* (E1F3-M)

In the family’s perception, individual or joint prayer was valued:

*Prayer is about strengthening and asking for more help so that we can be firm, to move on.* (E1F5-P)

As for the third category, that is, family resilience involving assertive communication, it was recognized that the difficulty that the family members experience rescued dialog between couples, with the development of "patience" being an apparent facilitator for union, as it would allow for evaluation and reflection of what occurs to them, to "tolerate" and "forgive".

*A little bit of dialog, which in the past was almost nonexistent. [...] Between me and him [couple].* (E1F2-M)

*Patience [...]. Patience is a very big fuel for unity. Whoever has no patience, does not move on. Patience gives you time to analyze the fact. If everything starts to explode right away, you don’t have it, you don’t have patience, then you don’t have time to analyze the fact: if I’m right, if I’m wrong, if I should tolerate, if she [wife] didn’t tolerate, but I will forgive, later we’ll talk about it.* (E1F5-P)

It was noticed, for example, the need to monitor the tone of the voice, so that there was mutual understanding and support.

*He [husband] is an example at home, I never lack anything, right? I always have his support in everything. Only, sometimes, I get a little nervous right? And sometimes I talk to him a little bit, he gets nervous, he talks to me loudly. Then I say: "We are yelling at each other". I said: "it can’t be". We try to correct ourselves [...] not getting angry at the other, not fighting, badly with each other, we’re always like this, leaning on each other.* (E1F4-M)

"Keeping calm" was also a strategy mentioned, allowing dialog and seeking solutions to problems, avoiding conflicts and aggressions.

*Because we [couple] can dialog, we can talk. If one gets out of their mind the other already speaks to take it easy, let’s talk, talk about the issue, look for a way to solve this issue, the person suggests the formula and the other analyzes and accepts. It is to impose a more stretched, calm conversation, with a more relaxed dialog. Mother gets angrier and right from the start she changes and the father doesn’t, father goes with other words, more calmly, seeking explanations, more in-depth, without allowing himself to be upset. Because when one gets upset, the other feels attacked and he also gets angry.* (E1F5-P)

## Discussion

In general, the literature highlights the predominance of the female figure in monitoring the treatment of PAS dependents, especially mothers, wives and grandmothers^(^ 14 ^)^. However, the data of the present study come from a sample that, although numerically reduced, was composed of a very close number of men and women (five and six, respectively); therefore, it presents balance from the point of view of family members in the perspective of gender.

Regarding the first category, it was observed that the participating families effectively and spontaneously mobilized in search of support and social support, reorganizing themselves to face the adversities, as occurs in other contexts already researched^(^ 7 ^,^ 15 ^-^ 16 ^)^.

The need to change the families’ routine to accompany the treatment of their sick member demonstrates the presence of one of the attributes of family resilience to solve problems, as it required a new standard of functioning to meet new family needs, corroborating the literature^(^ 6 ^)^. According to the systemic framework of family resilience, when dealing with an adverse situation, the family can mobilize resources and reorganize itself, adapting to the new demands derived from the changed living conditions. The family unit seeks to reorganize itself in a flexible and stable manner, in order to preserve its good functioning^(^ 6 ^)^.

The data in this study pointed to the search for a functional family balance in the work dimension, when a family member had to give up their job to take care of the dependent relative. The literature emphasizes that the attributes of family resilience involve reorganizing living conditions and often abandoning personal desires or other needs to adapt to the new demands, with a balance between daily work and household^(^ 6 ^)^. However, the problems experienced in the family environment can also result from specific social and economic contexts, and it seems important that health professionals consider these broader dimensions that can impact on the family resilience process. A study highlighted the need for the services to create strategies that facilitate the participation of the family members in the treatment of their relative, while avoiding the interruption of the work and entertainment routine^(^ 17 ^)^.

In the view of the participants in this study, cohesion and the affective and emotional connection between family members strengthen them to face and overcome crisis situations, having maintained, in their evaluations, some balance in the family unit. In fact, the interviewed family members hinted that they felt united in times of crisis, respecting the differences between them^(^ 6 ^)^. The sense of connection allows the family to work as a team^(^ 6 ^)^, establish flexible alliances, review individual roles and responsibilities, and achieve goals^(^ 4 ^)^. Family resilience is strengthened when the family members support each other, collaborate and commit to the process of facing the crisis^(^ 5 ^-^ 6 ^)^.

The participants expressed feeling the family group strengthened when experiencing the feeling of love permeating their relationships. In the same sense, a phenomenological study on the meaning of the family’s daily living with a member dependent on crack pointed out that, despite the emotional and physical overload that the family experiences, it manages to express feelings of love^(^ 5 ^)^. Loving tolerance and mutual support seemed to be present in the participants’ speeches, even when they experience possible contradictions (in F5-M’s speech, for example, "*we need to have love*", there seems to be a tension between a feeling immanent to the participant and a speech that carries certain formality).

In the interviews, mention was made of promoting intra-family and extended family life, which would have provided moments of leisure and distraction. The literature points out that family resilience benefits from such strategies, which favor family connection and cohesion^(^ 6 ^)^. In this way, the family would tend to perform better when balancing proximity and tolerance to differences^(^ 6 ^)^. Both in the intra-family environment and in the extended family, the moments of good mood and distraction seem to have been seen by the participants as factors that minimize distressing situations, possibly alleviating emotional overloads^(^ 5 ^-^ 6 ^)^, and favor the expression of emotions and feelings related to the adverse situation, something more difficult to occur in other contexts^(^ 5 ^)^.

Another attribute of family resilience identified by the family members in this study was support and extra-family support, involving community resources and the Psychosocial Care Network (Rede de Atenção Psicossocial, RAPS), which helped them to cope with the crisis and obtain informational, emotional and instrumental support. In fact, the family’s well-being and resilience in contexts of adversity depend on access to formal and informal support devices and resources^(^ 7 ^)^ and, in the opposite direction, on these devices transforming and adapting the family needs^(^ 7 ^)^. The CAPS-AD and its health professionals seem to have consisted of sources of informational and emotional support relevant to the respondents, particularly family groups. It is inferred that these families were able to recognize such relevance because, probably, such services enabled access and adapted to family demands. Such groups consisted, judging by the reports, of a welcoming space, listening and sharing experiences; being felt as promoters of mutual help, learning, re-elaboration of coping strategies, security and comfort. Thus, the family group seemed to strengthen family resilience, considering that group activities are usually recognized as a source of emotional support for the family members^(^ 18 ^)^, as the group’s demands would tend to be understood and welcomed without trials^(^ 17 ^)^.

Groups can create and strengthen bonds between the^(^ 1 ^,^ 17 ^)^ families and facilitate behavioral change through the development of patience, understanding and dialog^(^ 8 ^)^. Possibly, they instrumentalize care for the dependent family member^(^ 17 ^)^.

Friends were also recognized as a source of support and strength, corroborating the systemic theory of family resilience^(^ 6 ^)^, which emphasizes that social networks also involve community and religious groups and friends^(^ 7 ^)^, which can offer security and encouragement.

The second category explored beliefs that promoted positive and optimistic perspectives in the contexts experienced by the participants, favoring resilience. In fact, when the family experiences successful experiences and promotes a protective environment, the perspectives are revitalized and reinforced^(^ 6 ^)^.

Perseverance, persistence, hope, faith, religiousness and trust seemed to be constituted as positive forces for coping and overcoming difficulties arising from the context of dependence on PAS, usually permeated by negative and pessimistic emotions.

The hope that the family member will recover generates motivational feelings, which mobilize and strengthen the family members. Initiative and perseverance are fundamental elements for family resilience, often stimulated by shared trust^(^ 6 ^)^. Hope consists of the belief in a better future, regardless of the context that the family lives in the present, encourages in facing adversity and motivates the identification of positive alternatives^(^ 5 ^-^ 6 ^,^ 8 ^)^.

Families in difficult situations seek comfort and guidance when they connect spiritually with their cultural traditions, for example, spiritual and religious beliefs^(^ 6 ^,^ 8 ^)^.

For Christians, for example, and so all the research participants are considered, having faith and living spiritual experiences that they consider positive generate comfort^(^ 6 ^)^. Religious practices seem to constitute positive coping mechanisms about the life and treatment of people dependent on PAS^(^ 5 ^,^ 19 ^)^, as there would be a superior force that would help the family in overcoming crises^(^ 5 ^)^. In the sample, attending religious institutions, participating in their rituals, praying and meditating were the main sources of resilience considered; in these practices, the interviewees perceived relief and well-being, aspects that have been pointed out in other studies^(^ 4 ^,^ 6 ^)^. Family religious practices and beliefs seem to constitute mediating strategies for the health-disease process, as they promote resources for religious and spiritual coping and strengthen resilience^(^ 4 ^,^ 6 ^)^.

The third category pointed out that the families would have sought assertive communication to assist them in the resilience process, involving patient dialog, monitoring the tone of the voice, and maintaining calm.

Communication patterns strongly interfere in interpersonal relationships and can hinder or facilitate the management of the stressful situation^(^ 6 ^)^.

The adversity situation can promote family unity and connection, facilitating the sharing of feelings and open emotional expression^(^ 6 ^)^. However, the family that has the possibility to communicate in a thoughtful manner generally has better emotional conditions to experience the situation and solve their problems collectively^(^ 6 ^,^ 8 ^)^. The creation of spaces in the family context that facilitate emotional communication can promote resilience and minimize the negative impact in the face of adverse situations^(^ 20 ^-^ 21 ^)^.

An affective and optimistic tone of voice can facilitate the coping process, as the family members manage to demonstrate emerging feelings. Although the messages may be unexpected, they can be conveyed in an empathetic and respectful manner^(^ 6 ^)^.

Among the limitations of this study, there is the impossibility of validating the analyses with the family members, justified by the lack of time to find the research participants; therefore, the solution to minimize the impact of this limitation was to search the literature for evidence on aspects of resilience to discuss the results.

The contributions of this study refer to the use of the fundamental elements of resilience in the process of assessment and intervention in the care of families, which can help health and nursing professionals to recognize and value family strengths, helping them in the process of functional (re)organization. With this attitude, one can avoid stereotyping and labeling the family as "dysfunctional", "codependent" or "resistant", but also perceiving it as a potentially active agent in the process.

## Conclusion

This study made it possible to improve the understanding of some aspects of the perception of family members of PAS dependents about the elements of their functioning that help family resilience, going through their organizational patterns, their belief systems and their communication processes.

Although substance dependence has important repercussions on families, these adverse situations can also be seen as a challenge to be faced and overcome through the potentials and forces existing in the family system. Recognizing and strengthening these potentials seems important for the family to understand its role, as a unit capable of overcoming adversity and living healthier.
